# Supplementary material for: Characterizing steady states of genome-scale metabolic networks in continuous cell cultures
Source: PLoS Comput Biol. 2017 Nov 13;13(11):e1005835. doi: 10.1371/journal.pcbi.1005835 (PMC5703580; doi:10.1371/journal.pcbi.1005835)
Supplement: S1 Appendix — Contains mathematical derivations. (PDF) [file pcbi.1005835.s001.pdf]

Characterizing steady states of genome-scale metabolic networks in continuous cell cultures

Supporting Text

Jorge Fernandez-de-Cossio-Diaz (cossio@cim.sld.cu)   Kalet Leon

Roberto Mulet

October 14, 2017

1 Stability of fixed points

The growth rate μ depends on the biomass synthesis rate, z, and on the concentrations of toxic metabolites in the culture. Since for a fixed dilution rate, z depends only on X (cf. Eq. 6 in main text), it follows that we can write the growth rate as a function of X and $\underline{s}$, thus $\mu = \mu(X, \underline{s})$.

In particular note that the dynamics of non-toxic metabolites can be decoupled from the rest of the system (cf. equations 1 and 2 in main text). It is enough to determine the stability of a reduced system, where only X and the concentrations s_i of metabolites i that are toxic intervene. In the trivial case where there are no toxic metabolites all fixed points are stable because μ is a non-increasing function of X. We assume that $\partial\mu/\partial s_i < 0$ for all toxic metabolites i.

Let us begin by defining the velocities of change of X and s_i as the right-hand sides of Eq. 1 and 2 in the main text, respectively,

$$F(X, \underline{s}) = (\mu - \phi D)X \tag{1}$$

$$G_i(X, \underline{s}) = -u_i X - (s_i - c_i)D \tag{2}$$

A fixed point $\hat{X}, \hat{\underline{s}}$ satisfies $F(\hat{X}, \hat{\underline{s}}) = 0$ and $G_i(\hat{X}, \hat{\underline{s}}) = 0$. To determine its stability, we evaluate the Jacobian ($\mathbf{J}$) of equations 1 and 2 at $\hat{X}, \hat{\underline{s}}$:

$$\mathbf{J} = \begin{pmatrix} \frac{\partial F}{\partial X} & \frac{\partial F}{\partial s_i} \\ \frac{\partial G_j}{\partial X} & \frac{\partial G_j}{\partial s_i} \end{pmatrix} = \begin{pmatrix} \frac{\partial \mu}{\partial X}\hat{X} & -\frac{\partial \mu}{\partial s_1}\hat{X} & -\frac{\partial \mu}{\partial s_2}\hat{X} & \cdots & -\frac{\partial \mu}{\partial s_m}\hat{X} \\ -u_1\left(\hat{X}\right) - u_1'\left(\hat{X}\right)\hat{X} & -D & 0 & \cdots & 0 \\ -u_2\left(\hat{X}\right) - u_2'\left(\hat{X}\right)\hat{X} & 0 & -D & \cdots & 0 \\ \vdots & \vdots & \vdots & \ddots & \vdots \\ -u_m\left(\hat{X}\right) - u_m'\left(\hat{X}\right)\hat{X} & 0 & 0 & \cdots & -D \end{pmatrix} \tag{3}$$

where $u_i'(\hat{X})$ are the derivatives of $u_i(X)$ with respect to X, evaluated at the fixed point. To evaluate $\partial\mu/\partial X$, recall that $\mu = K \times z - \sigma$, where K and σ depend only on $\underline{s}$, while z is a function of X (at a fixed dilution rate, cf. Eq. 6 in the main text). Therefore, it is enough to evaluate $z'(X)$.

1.1 Computation of $z'(\hat{X})$ and $u_i'(\hat{X})$

Before continuing, let us make a short digression into Linear Programming [2]. To determine a *basic* solution of FBA, it is enough to specify: (i) the indexes of fluxes that are away from their lower and upper bounds, and (ii) for the remaining fluxes, whether they are equal to their lower or upper bound. This information is called the *basis* [2]. The full solution can be reconstructed from knowledge of the *basis* by solving the linear equality constrains. Since the basis is a discrete object, it will remain constant as ξ varies continuously, except for discrete 'critical' values of ξ where the basis changes. When the basis remains constant, $u_i^*(\xi)$ and $z^*(\xi)$ have the following forms:

$$u_i^*(\xi) = \alpha_i + \beta_i/\xi, \quad z^*(\xi) = \alpha + \beta/\xi, \tag{4}$$

where $\alpha_i, \beta_i, \alpha, \beta$ are constant as long as the basis remains fixed. Equation 4 is simply the generic affine dependency on the upper bounds of the uptakes (cf. Eq. 14 in main text). Since $z^*(\xi)$ is a non-increasing function of ξ, $\beta \geq 0$. Using Eq. 4, it follows that $u_i(\hat{X}) = \alpha_i + \beta_i D/\hat{X}$ and $z(\hat{X}) = \alpha + \beta D/\hat{X}$. Therefore:

$$\hat{u}_i + u_i'(\hat{X})\hat{X} = \alpha_i, \quad z'(\hat{X})\hat{X} = -\beta/\xi. \tag{5}$$

To obtain $\alpha, \beta, \alpha_i, \beta_i$, we exploit the fact that we will be computing $\mu^*(\xi) = z(X^*(\xi))$, $u_i^*(\xi) = u_i(X^*(\xi))$ and $X^*(\xi)$ over a sequence of contiguous values of ξ. If ξ_1, ξ_2 are sufficiently nearby:

$$\begin{aligned}\alpha_i &= \frac{u_i^*(\xi_1)\xi_1 - u_i^*(\xi_2)\xi_2}{\xi_1 - \xi_2}, \quad \beta_i = -\frac{u_i^*(\xi_1) - u_i(\xi_2)}{\xi_1 - \xi_2}\xi_1\xi_2, \\ \alpha &= \frac{z^*(\xi_1)\xi_1 - z^*(\xi_2)\xi_2}{\xi_1 - \xi_2}, \quad \beta = -\frac{z^*(\xi_1) - z^*(\xi_2)}{\xi_1 - \xi_2}\xi_1\xi_2.\end{aligned} \tag{6}$$

The singular case $\hat{X} = 0$ has $\beta = \beta_i = 0$, assuming that for very low cell densities growth is not limited by substrate availability (*i.e.*, that the medium is rich; cf. discussion before Eq. 6 of the main text).

From $z'(\hat{X})$ we compute $\partial\mu/\partial X = K \times z'(\hat{X})$.

1.2 Stability of the linearized system

The system is stable if the real parts of all the eigenvalues of $\mathbf{J}$ are negative, and is unstable if at least one eigenvalue has a positive real part [3]. The eigenvalues of $\mathbf{J}$ are:

$$\lambda_{\pm} = \frac{1}{2}\left(\mu'(\hat{X})\hat{X} - D \pm \sqrt{(D + \mu'(\hat{X})\hat{X})^2 + 4\hat{X}\omega}\right), \quad \lambda_{\mathrm{d}} = -D \tag{7}$$

where $\mu'(\hat{X})$ denotes the derivative $\partial\mu/\partial X$ evaluated at $\hat{X}$, and

$$\omega = -\sum_i \frac{\partial\mu}{\partial s_i}\left(\hat{u}_i + u_i'(\hat{X})\hat{X}\right) = -\sum_i \frac{\partial\mu}{\partial s_i}\alpha_i. \tag{8}$$

$\lambda_{\rm d}$ is a degenerate eigenvalue of order $m-1$ (where m is the number of metabolites) and is always negative (we assume that $D > 0$). The couple $\lambda_\pm$ forms a complex conjugate pair with negative real part if $(D + \mu'(\hat{X})\hat{X})^2 + 4\hat{X}\omega < 0$, which implies $\omega < 0 < \hat{X}$. In this case the system is stable. If $(D+\mu'(\hat{X})\hat{X})^2 + 4\hat{X}\omega \geq 0$ all the eigenvalues are real and all are negative except possibly λ_+. After some algebra, we find that $\lambda_+ < 0$ (the system is stable) or $\lambda_+ > 0$ (the system is unstable) according to whether $-\mu'(\hat{X})\hat{X} > \xi\omega$ or $-\mu'(\hat{X})\hat{X} < \xi\omega$, respectively. Since $\omega < 0 < \hat{X}$ implies $-\mu'(\hat{X})\hat{X} > \xi\omega$ (because $\mu'(\hat{X}) \leq 0$), the condition $-\mu'(\hat{X})\hat{X} > \xi\omega$ is sufficient for stability, while $-\mu'(\hat{X})\hat{X} < \xi\omega$ is sufficient for instability, even if $\lambda_\pm$ turn out to be complex.

The critical case $\lambda_+ = 0$ occurs whenever $-\mu'(\hat{X})\hat{X} = \xi\omega$. In this case the stability of the system cannot be resolved by analysis of the linearized system alone, and we must recur to the Center Manifold Theorem [1, Sec. 8.1]. As will be shown below, in this case the system is stable. Therefore, the fixed point is stable if $-\mu'(\hat{X})\hat{X} \geq \xi\omega$ and unstable if $-\mu'(\hat{X})\hat{X} < \xi\omega$. Since $\mu'(\hat{X})\hat{X}$ and ω are both independent of ϕ (by Eq. 5), this condition does not depend on ϕ. Then, whether a fixed point is stable or not can be given as a function of ξ only, as asserted in the main text.

The condition for stability can be further simplified by noting that $\mu'(\hat{X})\hat{X}/\xi + \omega$ is the derivative of $\mu^*(\xi)$ with respect to ξ. Therefore, the system is stable if an only if $\mu^*(\xi)$ is non-increasing in a neighborhood.

1.3 Center manifold stability for the critical case ($\lambda_+ = 0$)

If $-\mu'(\hat{X})\hat{X} = \xi\omega$ all eigenvalues are real and negative except $\lambda_+ = 0$. In this case the linearized system cannot be used to determine the stability of the fixed point, because the effect of small perturbations along the direction of the eigenvector corresponding to λ_+ (the so-called center manifold) is not captured by the linearized system. Since only one eigenvalue has a zero real part, the Center Manifold Theorem [1, Sec. 8.1] can be used to find a reduced one-dimensional system where the stability can be determined. For simplicity we will only consider the case where $\mu'(\hat{X}) = \alpha_i = 0$. The eigenvectors of $\mathbf{J}$ then are:

$$\underline{p}_1 = \begin{bmatrix} 1 \\ 0 \\ \vdots \\ 0 \end{bmatrix}, \quad \underline{p}_2 = \begin{bmatrix} -\xi\frac{\partial\mu}{\partial s_1} \\ 1 \\ 0 \\ \vdots \\ 0 \end{bmatrix}, \quad \underline{p}_3 = \begin{bmatrix} -\xi\frac{\partial\mu}{\partial s_2} \\ 0 \\ 1 \\ \vdots \\ 0 \end{bmatrix}, \quad \ldots, \underline{p}_{m+1} = \begin{bmatrix} -\xi\frac{\partial\mu}{\partial s_m} \\ 0 \\ 0 \\ \vdots \\ 1 \end{bmatrix} \tag{9}$$

where $\underline{p}_1$ corresponds to λ_+, $\underline{p}_2$ to λ_-, and the rest to λ_d. These eigenvectors are assembled into a similarity matrix $\mathbf{M}$ (as columns), which serves to diagonalize $\mathbf{J}$:

$$\mathbf{M}^{-1}\mathbf{J}\mathbf{M} = \begin{bmatrix} \lambda_+ & 0 & 0 & \cdots & 0 \\ 0 & \lambda_- & 0 & \cdots & 0 \\ 0 & 0 & \lambda_\mathrm{d} & \cdots & 0 \\ \vdots & \vdots & \vdots & \ddots & \vdots \\ 0 & 0 & 0 & \cdots & \lambda_\mathrm{d} \end{bmatrix} \tag{10}$$

Introduce new variables $x, z_1, \ldots, z_m$ through the relation:

$$\begin{bmatrix} X \\ \underline{s} \end{bmatrix} = \begin{bmatrix} \hat{X} \\ \hat{\underline{s}} \end{bmatrix} + \mathbf{M} \begin{bmatrix} x \\ \underline{z} \end{bmatrix} \tag{11}$$

To find the reduced system, we set $\underline{z} = 0$, which implies $X = \hat{X} + x$ and $\underline{s} = \hat{\underline{s}}$. Then, differentiating x with respect to time:

$$\frac{\mathrm{d}x}{\mathrm{d}t} = (\mu - \phi D)(\hat{X} + x) \tag{12}$$

The system is stable if and only if equation 12 is stable at $x = 0$. We show now that the right-hand side of equation 12 is a decreasing function of x, which implies stability. Since $s_i = \hat{s}_i$ is fixed, K, σ are constant. From Eq. 4 we know that $z = \alpha + \beta D/(\hat{X} + x)$ with constant α, β for sufficiently small x. Since $\hat{X}$ is a fixed point, it follows that $K(\alpha + \beta D/\hat{X}) = \sigma + \phi D$. Therefore the right-hand side of Eq. 12 is:

$$K\left(\alpha + \beta\frac{D}{\hat{X} + x} - \alpha - \beta\frac{D}{\hat{X}}\right)(\hat{X} + x) = -K\beta x/\xi \tag{13}$$

which is decreasing in x. This argument breaks down if $\beta = 0$, which occurs only in conditions of nutrient excess, where ξ is low enough that there is no nutrient competition between the cells. In this case $\beta_i = 0$ also for all i, implying that $\hat{u}_i = \alpha_i$ is piece-wise constant in this regime. If toxic metabolites are being secreted, $\omega > 0$ implying $-\mu'(\hat{X})\hat{X} = 0 < \xi\omega$, which falls under the umbrella of the non-critical linear stability analysis discussed above. If toxic metabolites are not being secreted, $\omega = 0$. But in the later case X is uncoupled from the rest of the variables, and the system is trivially stable because $\mu = \alpha$ is constant.

2 Alternative derivation of Equation 6 in the main text

An alternative uptake bound used in the literature is:

$$u_i \le \frac{V_i s_i}{K_i + s_i} \tag{14}$$

where V_i is the maximum uptake rate of metabolite i and K_i is the Michaelis-Menten constant. Eq. (6) in the manuscript can be derived as an approximation to equation 14. If a substrate is available in excess ($s_i \gg K_i$), this bound simplifies to $u_i \leq V_i$. In rich media at low cell densities this is the relevant regime. At higher cell-densities, substrates reach low levels ($s_i \ll K_i$), and the bound simplifies to $u_i \leq s_i V_i / K_i$. Employing the steady state metabolite concentration from Eq. (2), $s_i = c_i - u_i \xi$, we obtain $u_i \leq (c_i - u_i \xi) V_i / K_i$, or

$$u_i \leq \frac{c_i V_i / K_i}{1 + \xi V_i / X_i}$$

For high cell densities $\xi V_i / K_i \gg 1$, and the inequality simplifies to $u_i \leq c_i / \xi$. Combining the bounds obtained in both regimes leads to Eq. (6) in the main text. We prefer this form over Eq. 14 because it contains one less constant and simplifies mathematical derivations.

References

[1] Khalil HK. Nonlinear systems. 3rd ed. Prentice-Hall; 2002.

[2] Vanderbei RJ. Linear Programming. Foundations and Extensions. 4th ed. Springer; 2014.

[3] Strogatz SH. Nonlinear Dynamics and Chaos. Westview Press; 1994.
